# Autophagy: Guardian of Skin Barrier

**DOI:** 10.3390/biomedicines10081817

**Published:** 2022-07-28

**Authors:** Hyun Jee Kim, Jisoo Park, Sun Kyeon Kim, Hyungsun Park, Jung Eun Kim, Seongju Lee

**Affiliations:** 1 Department of Dermatology, Eunpyeong St. Mary’s Hospital, College of Medicine, The Catholic University of Korea, Seoul 03312, Korea; hyunjee0921@hanmail.net (H.J.K.); mdkjeun@naver.com (J.E.K.); 2 Program in Biomedical Science & Engineering, Inha University, Incheon 22212, Korea; jisoo630@inha.edu (J.P.); sunnykim@inha.edu (S.K.K.); hyungsun@inha.edu (H.P.); 3 Department of Anatomy, College of Medicine, Inha University, Incheon 22212, Korea

**Keywords:** autophagy, alopecia areata, psoriasis, atopic dermatitis, keloid, skin homeostasis

## Abstract

Autophagy is a major degradation pathway that removes harmful intracellular substances to maintain homeostasis. Various stressors, such as starvation and oxidative stress, upregulate autophagy, and the dysregulation of autophagy is associated with various human diseases, including cancer and skin diseases. The skin is the first defense barrier against external environmental hazards such as invading pathogens, ultraviolet rays, chemical toxins, and heat. Although the skin is exposed to various stressors that can activate autophagy, the roles of autophagy in the skin have not yet been fully elucidated. Accumulating evidence suggests that autophagy is closely associated with pathogenesis and the treatment of immune-related skin diseases. In this study, we review how autophagy interacts with skin cells, including keratinocytes and immune cells, enabling them to successfully perform their protective functions by eliminating pathogens and maintaining skin homeostasis. Furthermore, we discuss the implications of autophagy in immune-related skin diseases, such as alopecia areata, psoriasis, and atopic dermatitis, and suggest that a combination of autophagy modulators with conventional therapies may be a better strategy for the treatment of these diseases.

## 1. Introduction

Autophagy is a lysosome-mediated degradation system that removes intracellular materials, such as DNA, proteins, lipids, organelles, and invading pathogens [1]. Cells obtain energy by cannibalizing a part of themselves, in a process called “autophagy,” which means self-eating. Autophagy is an intrinsic defense mechanism that is essential for homeostasis and survival. Cells inevitably produce unnecessary proteins and dysfunctional organelles during their vital activities. Protein aggregates and dysfunctional organelles are harmful to both the cells and host; hence, cells eliminate them via autophagy. Additionally, cells can escape danger by performing autophagy, which degrades invading pathogens, such as microbes and viruses, during infection. Furthermore, cells acquire energy through autophagy during starvation, thereby maintaining homeostasis.

The skin is the first layer covering the animal body and serves as the first line of defense against external environmental hazards such as pathogens, allergens, ultraviolet rays, and various chemical toxins. As the immune system acts as the primary defense mechanism, the skin is rich in immune cells, which are either resident (Langerhans cells, macrophages, dermal dendritic cells, and mast cells) or recruited to the skin (neutrophils, natural killer (NK) cells, T cells, etc.) [2]. Immune cells collaborate with other cells, such as keratinocytes, in order for the skin to function as a barrier. The skin is a malnourished environment that is regularly exposed to several environmental stressors. The highly exposed and unfavorable circumstances of the skin lead to the activation of autophagy, which thereby allows the removal of external hazards and the maintenance of skin homeostasis. Thus, abnormalities in autophagy in the skin are closely linked to skin diseases. In this review, we briefly describe the molecular machinery of autophagy and discuss the roles of autophagy in skin cells, including keratinocytes and skin immune cells. Subsequently, we focus on elucidating the association of autophagy with immune-related skin diseases, including inflammatory diseases, infectious diseases, and malignant melanoma, proposing autophagy as a promising strategy for treating these diseases.

## 2. Molecular Machinery of Autophagy

**Figure 1:**
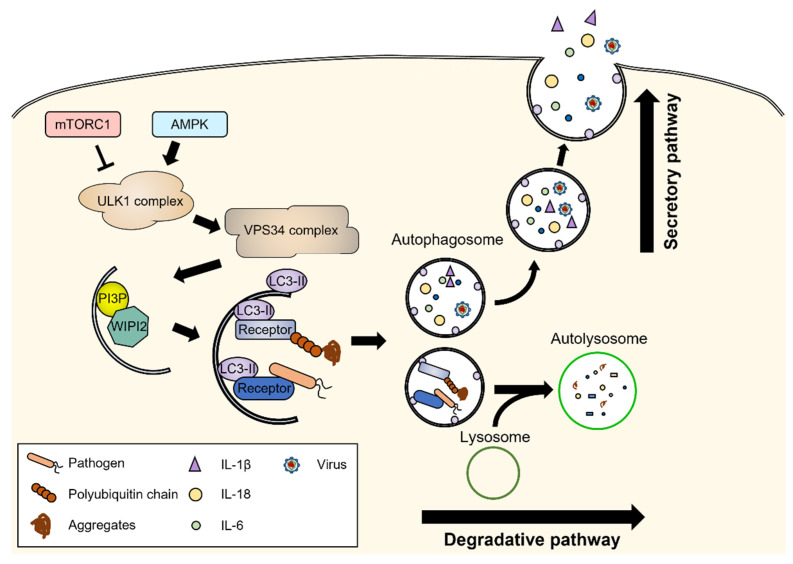
Outline of degradative and secretory autophagy pathways. Various stressors stimulate mTORC1 and AMPK, upstream signaling molecules of autophagy, which phosphorylate the ULK1 complex. The ULK1 complex sequentially activates the VPS34 complex, which generates PI3P at membrane sources and initiates autophagosome formation. With the aid of autophagic receptors, cargos are recruited to the autophagosomes. In the degradative autophagy pathway, the autophagosomes are fused with lysosomes, and their cargos are degraded by lysosomal enzymes. In the secretory autophagy pathway, autophagosomes escape the lysosomal degradation pathway and move to the cell periphery and fuse with the plasma membrane. The key inflammatory signaling molecules, such as interleukins, are released into the extracellular environment. Detailed mechanisms are described in the text.

In mammals, autophagy is classified into three types according to the cargo delivery mechanism to lysosomes: chaperone-mediated autophagy (CMA), microautophagy, and macroautophagy [3,4]. In CMA, as the name suggests, proteins harboring the KFERQ motif are identified by the chaperone and transported into lysosomes via the oligomeric LAMP2A protein channel. In microautophagy, which is less studied than the other forms, cargos or proteins with KFERQ are engulfed directly or via endosomes into lysosomes. Macroautophagy, hereafter referred to as autophagy, is characterized by double-membrane vesicles called autophagosomes that sequester cargo and fuse with lysosomes for degradation (Figure 1). As the process is highly dynamic, autophagic flux is assessed to accurately monitor autophagic degradation activity. Autophagic flux is measured as the rate of degradation of autophagosomal marker proteins after blocking autophagic degradation [5]. Defects in autophagic flux have been reported to be associated with various pathological conditions, such as neurodegeneration and immune-related diseases [6]. Autophagy can degrade cargo in bulk or selectively through autophagic receptors. Autophagic receptors directly bind to specific cargo and light chain 3 (LC3), a representative autophagosomal membrane protein, thereby recruiting cargo to the autophagosomes. For example, p62 binds to ubiquitinated proteins and mediates their degradation; this process is called aggrephagy [7]. Additionally, autophagic adaptors recognize specific cargos. Moreover, autophagic adaptors interact with autophagy-related proteins to serve as scaffolds for autophagic degradation [8]. Tripartite motif (TRIM) proteins, such as TRIM5α and TRIM20, interact with cargo and core autophagy regulators to form protein complexes called “TRIMosomes”.

Autophagy is tightly regulated by autophagy-related proteins and occurs sequentially, including during initiation, autophagosome formation, fusion with lysosomes, and autophagic lysosomal reformation [3,9]. The mammalian target of rapamycin complex 1 (mTORC1) inhibits autophagy in the basal state. Various cellular stressors, such as starvation and hypoxia, inhibit mTORC1, thereby inducing the initiation of autophagy. The initiation of autophagy requires the activation of Unc-51-like autophagy-activating kinase 1 (ULK1), which is regulated by mTORC1 and AMP-activated protein kinase (AMPK). ULK1 interacts with FIP200, ATG101, and ATG13 to form the ULK1 complex. The ULK1 complex phosphorylates the class III phosphoinositide 3-kinase (PI3K) complex consisting of VPS34, VPS15, ATG14L, and Beclin-1. The PI3K complex generates phosphatidylinositol 3-phosphate (PI3P) at membrane sources; thus, it becomes a phagophore and recruits PI3P-binding proteins such as WD repeat domain phosphoinositide-interacting protein 2 (WIPI2). During phagophore elongation, several ATG proteins convert LC3 to phosphatidylethanolamine-conjugated-LC3 on the phagophore via the ubiquitin-like conjugation system [10]. Simultaneously, ATG9 supplies phospholipids for phagophore elongation [11]. Elongating phagophores sequester cargo and form autophagosomes after their closure. The autophagosome is translocated close to lysosomes via motor proteins and fuses with lysosomes through the soluble N-ethylmaleimide-sensitive factor attachment protein receptors (SNARE) complex [12]. In autolysosomes, the cargo is degraded by lysosomal enzymes. After lysosomal degradation, tubular structures stretch from autolysosomes and become proto-lysosomes to restore free lysosomes; this is called autophagic lysosomal reformation and is the terminal step in autophagy [13].

Although autophagy is generally thought to be a degradative process, recent studies have revealed that it participates in the extracellular secretion of cytoplasmic materials that do not enter the conventional secretory pathway through the Golgi apparatus. This unconventional secretion pathway is known as secretory autophagy (Figure 1). During secretory autophagy, autophagosomes escape the lysosomal degradation pathway and fuse with the plasma membrane to be released into the extracellular environment [14]. Secretory autophagy seems to be strongly associated with the immune response because representative proteins released by secretory autophagy are interleukin (IL)-1β, IL-18, and IL-6, which are the key inflammatory signaling molecules in the immune system [15]. In contrast, viruses employ secretory autophagy for subsequent infection. After host cell infection, poliovirus and influenza A virus spread into the intracellular environment through secretory autophagy and invade other cells [16]. Consequently, degradative autophagy and secretory autophagy contribute to defending cells from harmful external factors.

## 3. Autophagy in Keratinocytes

Keratinocytes are the primary cell type found in the epidermis, accounting for 90% of the epidermis, and are composed of highly keratinized squamous cells. During the differentiation of keratinocytes, they lose their division ability, nucleus, and cytoplasmic organelles, and migrate towards the surface, eventually becoming corneocytes in the stratum corneum, the outermost layer of the skin. Thus, keratinocytes act as the first barrier protecting the skin from external environmental hazards such as UV radiation, wounds, water loss, and infection [17]. Autophagy plays a critical role in keratinocyte differentiation and its function as a skin barrier.

**Figure 2:**
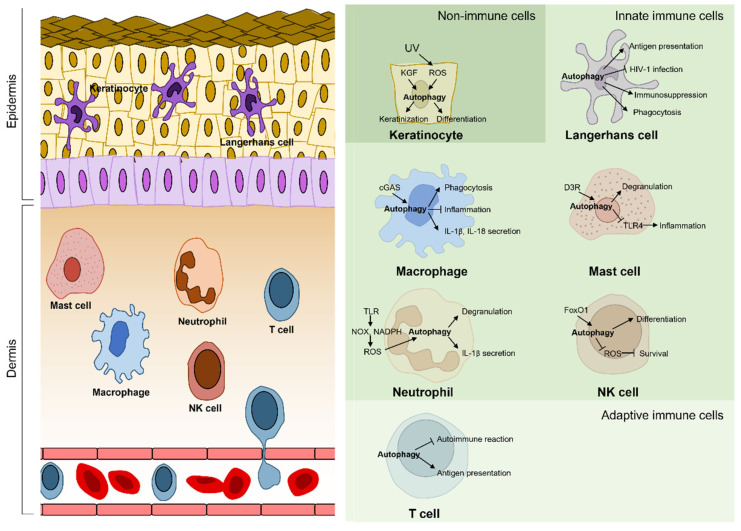
Population of immune-related cells in the skin. Keratinocytes are the main component of the epidermis. In the process of acting as the first skin barrier, keratinocytes also participate in immune-related responses. Under normal conditions, Langerhans cells, macrophages, mast cells, and T cells constitute the skin-resident immune system, which induces a rapid innate response. Upon activation of the immune system, additional immune cells, such as neutrophils, NK cells, and T cells, are recruited to the skin to aid the immune responses. Autophagy occurs actively in the skin because numerous stressors that activate autophagy are applied to the skin. Autophagy supports the skin barrier by mediating the function of the immune-related cells. Cell-type-specific pathways are described in detail in the text and figure.

Autophagy occurs continuously and actively in the epidermal layer [18]. Various stresses applied to the epidermis can act as triggers to activate autophagy, which contributes to keratinocyte differentiation (Figure 2). For example, mitochondrial reactive oxygen species (ROS) produced during oxidative phosphorylation and released into the cytoplasm in suprabasal keratinocytes trigger autophagy, which is necessary for epidermal differentiation [19]. The keratinocyte growth factor (FGF7/KGF) was observed to induce autophagy via the PI3K-AKT-mTOR pathway, which promoted keratinocyte differentiation [20]. Inhibiting autophagy by depleting ATG5, ATG7 or Beclin-1 suppressed the proliferation and differentiation of keratinocytes [19,21]. In particular, autophagy functions in keratinocyte differentiation via nuclear removal. During the terminal differentiation of keratinocytes, autophagosomal proteins, such as LC3, p62, and ULK1, and the lysosomal protein LAMP2 showed perinuclear localization and the nuclei became irregular and malformed [18]. The selective autophagic degradation of nuclear materials is called nucleophagy [22]. Atg5- or Atg7-deficient keratinocytes showed increased DNA damage, cellular senescence, and an aberrant lipid composition [23,24].

Secondly, autophagy supports function of keratinocytes in the skin barrier. As external hazards encountered by the skin are significant inducers of autophagy, autophagy generally responds by clearing them and triggering an inflammatory response. More directly, autophagy regulates the expression of keratinocyte proteins involved in barrier function. In a previous study, skin grafts from Atg7-deficient mice displayed acanthosis, hyperkeratosis, and abnormal hair growth [25]. The expression of keratinization-related proteins, such as loricrin, filaggrin, and involucrin, was reduced in the mice. Additionally, mislocalization of integrin α6 was observed in the epidermis of keratinocyte-specific Beclin-1 knock-out mice, which eventually died because of severe damage to the epidermal barrier [26].

Wound healing begins after pathogen invasion or epidermal layer injury. During wound healing, keratinocytes interact with fibroblasts and result in complex processes, including hemostasis, inflammation, proliferation, and remodeling. Autophagy promotes wound healing by modulating the TNF-CCL2 pathway, which coordinates keratinocyte–fibroblast interactions [27]. In addition, autophagy promotes the secretion of vascular endothelial growth factor (VEGF) from mesenchymal stem cells through direct extracellular signal-regulated kinase (ERK) phosphorylation during wound healing [28]. In addition, autophagy is involved in NLRP3-inflammasome activation by mediating Notch1 degradation in keloids [29,30].

## 4. Autophagy in Skin Immune Cells

### 4.1. Langerhans Cells

Langerhans cells (LCs) are tissue-resident macrophages, which are specialized dendritic cells that present antigens for T cells in the epidermis [31]. LCs can probe antigens from the external environment through their dendrites that come into contact with the stratum corneum. In the case of skin infection, LCs phagocytize and process pathogens and present them on major histocompatibility complex (MHC) class II molecules to induce the immune response of T helper cells [31,32]. In human LCs infected with *Mycobacterium leprae* (*M. leprae*), autophagy enhances the antigen presentation ability of LCs infected with *M. leprae* (Figure 2) [33]. In addition, LCs express Langerin, an HIV-1 receptor, and restrict HIV-1 infection via a TRIM5α-mediated mechanism. After HIV-1 exposure, Langerin in LCs interacts with Atg16L1 and TRIM5α, well-known autophagic adaptors, which leads to autophagic degradation of the Langerin-HIV-1 capsid complex [34]. In addition to canonical autophagy, noncanonical autophagy contributes to the immunosuppression of LCs in the epidermis. Rubicon, which facilitates VPS34 activity and stabilizes the NOX2 complex for ROS production, is critical for the progression of LC3-associated phagocytosis, a type of noncanonical autophagy [35]. The skin of Rubicon-deficient mice shows hyper-sensitive inflammation in UV-induced immunosuppression [36]. Collectively, autophagy is essential for LCs to conduct antigen presentation, pathogen removal, and immunosuppression, which contributes to the maintenance of immune homeosis of the epidermis.

### 4.2. Macrophages

Macrophages are essential immune cells that are responsible for inflammation and wound healing. Macrophages engulf and degrade pathogens through phagocytosis as white blood cells. In particular, macrophages in the skin are specialized to engulf melanosomes or melanocyte debris, known as melanophages. Like neutrophils, macrophages digest phagosomes using autophagic machinery triggered via Toll-like receptor (TLR) signaling (Figure 2) [37]. However, some bacteria, such as *Mycobacterium tuberculosis*, can escape from phagosomes and avoid degradation [38]. Even so, macrophages can detect bacteria using a cytosolic DNA sensor, cyclic GMP-AMP synthase (cGAS), and activate xenophagy to kill them selectively [39]. Additionally, autophagy is associated with inflammatory signaling pathways in macrophages. Macrophages degrade apoptosis-associated speck-like protein containing a CARD (ASC), a part of the inflammasome, via autophagy to inhibit inflammasome activation and increase the secretion of IL-1β or IL-18 [40,41]. Incidentally, melanophages consume melanosomes and process LC3-associated machinery [42]. This is essential for skin homeostasis as melanin protects skin cells from UV radiation and toxic metal ions [43,44]. Collectively, macrophages can achieve skin homeostasis, immune defense, and signal activation through autophagy.

### 4.3. Mast Cells

Mast cells are a type of granulocyte that are critical for allergic inflammatory reactions. Mast cells contain many granules filled with mediators, such as histamine, heparin, and chymase. Mast cells release inflammatory mediators from the granules into the extracellular space and activate allergic responses upon activation. The degranulation of mast cells requires autophagic machinery (Figure 2). In a previous study, autophagy was induced in mast cells even under full nutritional conditions and LC3-II was associated with secretory granules [45]. Atg7-deficient mast cells exhibited impaired degranulation and a poor response to passive cutaneous anaphylactic reactions [45]. Moreover, the dopamine D3 receptor (D3R) of mast cells induced the autophagic degradation of TLR4, thereby preventing excessive inflammation [46]. These studies imply that further research on the autophagy of mast cells may provide a key mechanism for hypersensitivity.

### 4.4. Neutrophils

Neutrophils are the most abundant leukocytes, accounting for approximately 50–70% of white blood cells in the body. However, neutrophils are rarely found in healthy skin. Under inflammatory conditions, they are recruited to the skin and enriched. As soldiers of the immune system, neutrophils are responsible for a variety of functions, such as pathogen phagocytosis, degranulation, ROS production, neutrophil extracellular trap (NET) formation, and IL-1β secretion [32]. In these processes, autophagy supports neutrophils (Figure 2). During phagocytosis, TLR signaling initiates the recruitment of LC3 to phagosomes, followed by the autophagic degradation of pathogens [47]. TLR signaling activates NOX2 NADPH oxidase, which generates ROS, a prerequisite for LC3-associated phagocytosis [37]. Conversely, autophagy-deficient mice exhibited reduced NADPH-mediated ROS production [48]. In addition, neutrophils require autophagy for degranulation, the secretion of antimicrobial cytotoxic agents, and other functions. Neutrophils from Atg5- or Atg7-deficient mice failed to mediate inflammation, owing to reduced degranulation [48]. In addition, autophagy mediates IL-1β secretion. Inhibition of autophagy by siRNA-mediated knockdown or drug treatment in human neutrophils significantly decreased IL-1β secretion via the unconventional secretory autophagy pathway [49]. However, the effect of autophagy on NET formation, composed of DNA from neutrophils, is still unclear. Although NET formation largely depends on ROS generation, some studies have reported ROS-independent NET formation [50,51]. In a previous study, during pneumoseptic infection, neutrophils from mice deficient in Mincle, a C-type lectin receptor, showed attenuated NET formation and impaired autophagy, regardless of ROS production [52]. Treatment of the mice with an autophagy inducer rescued the NET formation defect, which suggests that Mincle regulates NET formation via autophagy [52]. In contrast, another study reported that Atg5-deficient neutrophils did not affect NET formation [53]. Altogether, neutrophils require autophagy for phagosome degradation and the activation of their signaling pathways.

### 4.5. NK Cells

NK cells are cytotoxic lymphocytes that are critical for the innate immune system and allow for a faster immune reaction because they recognize target cells without MHC. Autophagy plays a crucial role in NK cell development (Figure 2). Robust autophagy occurs in immature NK (iNK) cells, and *Atg5*-deficient NK progenitors cannot become iNKs or NKs [54,55]. After a viral infection induces mitochondrial defects in NKs, forkhead box O (FoxO) 1-induced autophagy removed damaged mitochondria and ROS, thereby enabling NK cell survival and NK-cell-mediated innate immunity [54]. The severity of a viral infection on the skin is determined by the primary responses, mediated mainly by NK cells, with consequences ranging from flares to cancer [56]. NK cells secrete small granules containing perforin and granzymes to kill target cells. In melanoma cells, Beclin-1 inhibition prevented Granzyme B degradation and increased NK cell infiltration in a CCL5-dependent manner, thereby inhibiting melanoma growth [57,58]. Therefore, autophagy is essential for the development and immune response of NK cells.

### 4.6. T Cells

Human skin contains approximately 20 billion T cells, nearly twice the number in the whole blood [59]. T cells are essential in adaptive immunity and are distinguished from other lymphocytes by their unique functional T cell receptors. Skin resident-memory T_reg_ cells relieve inflammation and regulate immune responses through autophagy (Figure 2). Atg7-deficient T_reg_ cells lost skin homeostasis, causing skin diseases such as autoimmunity, allergies, and malignancy [60]. In autophagy-defective dendritic cells, antigen presentation and CD4^+^ cell responses were severely impaired [61]. In addition, Foxp3^+^ T_reg_ cells suppressed autoimmune responses by inhibiting the machinery of autophagy. Furthermore, treatment with roseotoxin B induced autophagy in activated T cells, which resulted in the mitigation of allergic contact dermatitis in mice [62]. As such, autophagy plays an essential role in regulating the immune response of T cells in the skin barrier.

## 5. Autophagy in Inflammatory Skin Diseases

### 5.1. Alopecia Areata

Alopecia areata (AA) is an inflammatory and autoimmune disease characterized by a non-scarring alopecic patch or patches that may extend to the entire scalp (alopecia totalis) or even the entire body (alopecia universalis) [63]. When a collapse in the immune privilege of hair follicles (HFs) develops, HFs present surface autoantigens and cause inflammatory cells to attack HFs, eventually resulting in AA. Current treatments for AA include corticosteroids, topical minoxidil, anthralin, cyclosporine, photochemotherapy, contact immunotherapy, and targeted immune therapy. However, achieving a complete response without relapse remains challenging.

Regulated cell death, including apoptosis, necroptosis, and autophagy, is a central element in the progression and regression of each HF cycle [64]. In AA, abnormal coordination of HF cycling has been reported [65]. Transcriptional profiling of gene expression for T-cell mediated immune responses and cell proliferation arrest was affected in patients with AA. Autophagy has been shown to be critical in maintaining active hair growth (anagen) during the hair cycle [66]. Additionally, autophagy promotes the differentiation of hair follicle stem cells [67].

The role of autophagy in AA pathogenesis has recently been studied at a molecular level. The first genome-wide meta-analysis on AA patients revealed significant associations of the autophagy-regulatory genes STX17 and BCL2L1 with AA [68]. Decreased expression of ATG4B in scalp biopsies of AA patients has also been reported [69]. Another study reported changes in autophagy-related protein levels in occipital scalp HFs obtained from patients with active AA [70]. HFs of lesional AA displayed reduced levels of the autophagy markers ATG5 and LC3B in the hair matrix. Conversely, the protein level of p62, which is degraded within active autolysosomes, significantly increased, thereby indicating a reduction in intrafollicular autophagy during AA progression. Hair-growth-promoting nutraceuticals, such as (methyl-)spermidine, partially restored intrafollicular autophagy, which suggests that they may be considered as adjunct therapies in the future management of AA. The role of autophagy in AA pathogenesis and its potential as a therapeutic target in AA warrants further study.

### 5.2. Psoriasis and Atopic Dermatitis

Psoriasis (PS) and atopic dermatitis (AD) are chronic inflammatory skin diseases characterized by a dysfunctional epidermal barrier and an excessively activated immune system. PS is strongly associated with dysfunctional helper T cells (Th1, Th17, Th22, and Treg) and is characterized by sharply delineated scaly erythematous plaques. In plaque PS, the intricate inflammatory cascade mediated by the IL-23/IL-17 pathway acts on keratinocytes, endothelial cells, and immune cells, thereby stimulating epidermal hyperplasia and the pro-inflammatory feed-forward circuit [71]. In addition, the function of Tregs, which play a fundamental role in immune homeostasis by suppressing immune responses, appears to be impaired in PS, which leads to an altered ratio of Th17 to Treg and exacerbation of the disease [72]. AD is driven primarily by differentiation defects of terminal keratinocytes and strong activation of Th2 immune responses. AD is characterized by impaired skin barrier function, pruritus, and a close association with immunoglobulin-E-mediated sensitization to aeroallergens and foods [73,74]. Like PS, AD is considered a primarily T-cell-driven disease but differs from PS in that Th22, Th17/IL-23, and Th1 cytokine pathways are additionally activated in AD [73]. Additionally, a significant increase in B cells in the blood is observed in AD, perhaps reflecting atopic or allergic associations and atopic march [73].

Accumulating evidence has shown that autophagy is involved in PS and AD; however, the activities of autophagy in PS and AD are controversial. Recently, increased Beclin-1 expression in the skin of patients with PS has been reported [75]. Additionally, significant increases in oxidative stress and autophagy-related proteins were observed in the blood cells of PS patients [76]. These results suggest that increased autophagy in response to oxidative stress, favoring NF-κB activation, may enhance the production of pro-inflammatory cytokines in patients with PS, and the use of antioxidant compounds may be beneficial in the treatment of PS.

In contrast, several studies have shown that autophagy is dysfunctional in AD and PS. In epithelial cells obtained from patients with AD and PS, the expression levels of ATG5, ATG7, and ATG8/LC3 were increased, which may indicate the induction of autophagy. On the other hand, p62, which is degraded by autophagy, was present at elevated levels in both AD and PS, which suggests the inhibition of autophagy [77]. This result indicates that autophagic degradation is impaired despite increased autophagosome formation [30,78]. Indeed, an in vitro study showed that lysosomal enzymes, such as cathepsins, were downregulated in skin specimens from AD and PS patients and keratinocytes treated long-term with TNF-α [77]. This indicates a functional impairment of autophagic degradation in AD and PS, which suggests that long-term exposure to TNF-α suppresses autophagy and reduces the fusion of autophagosomes with lysosomes. Impaired autophagy in AD and PS could result in reduced keratinocyte differentiation, epithelial barrier dysfunction, and enhanced inflammation [18], which suggests that the stimulation of autophagy might be a novel treatment option for these diseases. In line with this, IL-37 was shown to ameliorate allergic inflammation by regulating the microbiota and stimulating the mTOR-dependent autophagy signaling pathway in a mouse model of AD [79]. IL-37 downregulated the expression levels of mTOR by increasing AMPK levels, thereby leading to enhanced autophagy and a reduction in the expression of inflammatory cytokines such as IL-31 and IL-33. The digestion of dysfunctional mitochondria by autophagy prevented the release of mitochondrial ROS, which is known to activate inflammasome complexes and produce IL-1β [80], thereby resulting in a decrease in pro-inflammatory cytokine maturation. Moreover, autophagy prevents the cleavage of pro-IL-1β into its active form [41,81]. In the clinical setting, treatment with a moisturizer containing pentasodium tetracarboxymethyl palmitoyl dipeptide-12 (PTPD-12), a known autophagy stimulator, improved skin barrier function and pruritus by controlling inflammation in patients with AD [82]. The anti-inflammatory effect of autophagy through the control of IL-1β-induced inflammation explains the improvement in pruritic symptoms in AD patients treated with PTPD-12.

Some genetic associations between skin inflammation and autophagy have been reported. AP1S3, a gene implicated in autophagosome formation, was found to be mutated in PS patients [83]. Keratinocytes lacking AP1S3 exhibited defective autophagy and p62 accumulation and promoted skin inflammation as a consequence of increased NF-κB activation and secretion of IL-1β and IL-36 [84]. The association of polymorphisms in ATG16L1, a representative autophagy regulator, with PS has also been identified in Estonian patients [85]. This is interesting because several earlier studies also identified ATG16L1 as a susceptibility gene in Crohn’s disease and ankylosing spondylitis, thereby indicating that defective autophagy might be a general feature contributing to the pathogenesis of autoinflammatory diseases.

The dominant effects of autophagy modulation on keratinocytes and immune cells in different pathological condition should be carefully evaluated to identify autophagy modulators as potential candidates for the treatment of inflammatory skin diseases.

### 5.3. Keloids

A keloid is a chronic inflammatory fibroproliferative condition over an original epithelial injury that arises from an imbalance between the increased synthesis of extracellular matrix proteins, such as collagen, and the decreased degradation of these proteins [86]. Inflammatory mediators, such as TGF-β, have been proposed to influence the dysregulation of collagen remodeling in the scar-healing process. Therapies, such as steroids, radiation, cryotherapy, and lasers, are used for treatment, but keloids still have a high recurrence rate [86]. Numerous studies have suggested autophagy modulators as novel treatments for keloids.

A genome-wide association study with keloid patients in the Japanese population revealed an association between keloids and NEDD4, an E3 ubiquitin ligase acting as a positive autophagy regulator, thereby implicating the potential roles of autophagy in the pathogenesis of keloids [87]. Recent studies have shown increased levels of autophagy proteins in keloid tissues. An in vitro study by Jeon et al. reported increased levels of Beclin-1, LC3, and HMGB1, an exogenous fibrogenic molecule in keloid tissues [88]. Inhibition of HMGB1 with glycyrrhizin reduced ECM expression and autophagy in keloids but enhanced apoptosis, thereby suggesting its potential use in keloid treatment. Additionally, two other studies using skin specimens from keloid patients have reported increased LC3 expression in keloid fibroblasts [89,90]. Both studies suggested that hypoxia, a major characteristic of keloids, increases autophagy in keloids, as hypoxia is a positive signal for autophagy. These results suggest that the inhibition of autophagy may have therapeutic implications.

Contrary to these findings, our previous in vitro study demonstrated that autophagy is disturbed in keloid fibroblasts, leading to the activation of Notch1-mediated NLRP3 inflammasome signaling, which is critical for chronic inflammation [30]. Though protein levels of LC3 were increased, autophagic flux was significantly reduced in keloid fibroblasts. Due to aberrant autophagic activity, the levels of Notch1, which is degraded by autophagy [29], are elevated in keloid fibroblasts, which induces NLRP inflammasome activation. In addition, our study showed that rapamycin, an mTOR inhibitor and a well-known autophagy inducer, decreased the protein levels of Notch1, the NLRP3 inflammasome complex, and TGF-β3 in keloid fibroblasts. These results suggest that the induction of autophagy with rapamycin can attenuate keloidal fibrosis by downregulating the Notch1-NLRP3 inflammasome pathway and myofibroblast differentiation in keloid fibroblasts. This finding is consistent with the results of a study that reported that rapamycin has therapeutic efficacy in keloids [91]. The complicated role of autophagy in keloids requires further investigation.

## 6. Autophagy in Infectious Skin Diseases

The skin is a barrier that serves as one of the body’s first lines of defense against foreign pathogens through a variety of processes. When a breach of the skin barrier allows pathogens to enter the site of injury, the innate immune system and its effectors play a role in protecting the body from infection. Major constituents of the innate immune system include phagocytic cells, such as macrophages, neutrophils, and dendritic cells, as well as innate leukocytes, such as NK cells, mast cells, basophils, and eosinophils [92]. Furthermore, autophagy is an essential defense mechanism in the skin against invading pathogens. However, the role of autophagy in infectious skin diseases may differ depending on the pathogen type, infection stage, and microbial strain. Therefore, the role of autophagy in infectious skin diseases should be considered in the context of infection, and we should be aware that autophagy can be manipulated in both the host and the pathogen.

Most viruses have been reported to be related to autophagy, which functions as either an antiviral pathway (that degrades viruses) or a pro-viral pathway (that helps viruses replicate or exit from cells), and as a pathway that regulates innate and adaptive immune responses to viral infections [93]. Autophagy is triggered by viral ligands for pattern recognition receptors and cytokines, thereby limiting viral replication through the degradation of viral particles [94]. Varicella-zoster virus (VZV) and herpes simplex virus-1 (HSV-1) belong to the same family of alpha-herpesviruses, but their interactions with the autophagy pathway are very different. HSV-1 causes a lifelong latent infection in sensory ganglia neurons and is periodically reactivated to induce recurrent lesions in the skin and mucosae [95]. When reactivation occurs, the virus travels back along the axons to the primary infection site, where a new round of replication, a recurrent or secondary infection, begins. During this process, new neuronal infections occur. The HSV-1 neurovirulence factor ICP34.5 directly targets Beclin-1 and inhibits Beclin-1 complex formation [96]. In addition, ICP34.5 inhibits TBK1 and prevents cargo recruitment via TBK1-mediated p62 phosphorylation. US11, a viral tegument protein, also inhibits autophagy by inhibiting eukaryotic translation initiation factor 2 α kinase 2 (EIF2AK2). The inhibitory effects of ICP34.5 and US11 on autophagy allow for the more efficient production of viral progeny by maintaining low levels of viral protein degradation. Therefore, neuronal autophagy regulatory proteins may be involved in HSV-1 pathogenesis.

VZV causes varicella (chickenpox) as a primary infection, and VZV reactivation after several years of latency in neurons in the peripheral ganglia causes herpes zoster (shingles), a painful vesicular rash occurring within the distribution of a specific sensory dermatome [97]. VZV lacks the genes encoding ICP34.5 or US11 that interfere with autophagy in HSV-1 infection. Nonetheless, a high prevalence of VZV suggests that the absence of these proteins does not significantly impair the virulence of VZV and suggests a different mode of interaction between VZV and autophagy. Studies have demonstrated both the pro-and anti-viral roles of autophagy in VZV immunity and pathogenesis. Although autophagy appears to exert antiviral roles in some contexts, the virus may also directly utilize the machinery of autophagy for its own benefit to enhance viral replication and egress, thus evading autophagy and subverting this process [97].

One of the most studied skin pathogens related to autophagy is group A streptococcus (GAS), one of the most common causative organisms of skin and soft tissue infections, along with *Staphylococcus aureus* (*S. aureus*). GAS and *S. aureus* are associated with a wide range of skin diseases, which include impetigo, cellulitis, and toxic shock syndrome. Impetigo is a pustular infection of the epidermis in which bacteria infect the host through a break in the skin. Cellulitis is an acute infection of dermal and subcutaneous tissues that occurs after the protective integrity of the epidermis is compromised, through processes such as trauma, ulcers, and eczema, allowing bacteria to gain access to the subepidermal tissues [98]. Toxic shock syndrome is a life-threatening disease caused by superantigens of *S. aureus* or GAS. It bypasses normal antigen presentation and causes clonal T-cell expansion and the uncontrolled release of pro-inflammatory mediators, leading to severe multiple organ failure [99]. Due to bacterial evolution and antibiotic abuse, the drug resistance of GAS and *S. aureus* has gradually increased, and treatment has become more difficult. An in vitro study by Nakagawa et al. first showed that autophagy is important for defense against bacterial pathogens that invade the cytosol, such as GAS [100]. They found that cytoplasmic GAS that escaped from endosomes was engulfed by autophagosomes and killed upon the fusion of autophagosomes with lysosomes. Another in vitro study showed that the autophagy receptor Tollip facilitated bacterial autophagy by recruiting galectin-1 and -7 in response to GAS infection [101]. In addition, low pH seemed to play a significant role in GAS removal via autophagy because insufficient acidification of autophagosomes permitted GAS replication in vitro, which led to the growth of GAS in endothelial cells [102]. However, another in vitro study showed that GAS has evolved mechanisms to avoid autophagic degradation by employing streptococcal pyrogenic exotoxin B (SpeB1) to induce the degradation of p62, NDP52, and NBR1, thereby resulting in the escape of GAS from host autophagy [103].

The emergence of antibiotic-resistant strains of bacteria, such as methicillin-resistant *Staphylococcus aureus* (MRSA), has led to the need for new therapies, such as autophagy modulators, that can target intracellular pathogens. A previous in vivo study using mouse models showed that vancomycin encapsulated within liposomes was efficiently taken up by Kupffer cells and killed intracellular *S. aureus* [104]. Additionally, autophagy modulators were suggested to be combined with liposomes to minimize side effects on the host, such as facilitating other bacterial infections [105].

Nontuberculous mycobacteria (NTM) are another causal pathogen of the skin. Cutaneous NTM infections are transmitted via direct inoculation through skin barrier breaks, which can occur during trauma, surgical procedures, injections, tattoos, acupuncture, and body piercings [106]. NTM skin infections usually require surgical debridement, along with a combination of antibiotic therapy that should be administered long enough to ensure complete wound healing and prevent recurrence [107]. Despite the use of multidrug regimens, antibiotic resistance of NTM is an emerging problem. The data implicating host autophagy in infection with NTM bacteria strongly suggest that autophagy-activating agents are potential candidates for host-directed therapeutics during NTM infection [108].

## 7. Autophagy in Malignant Melanoma

Malignant melanoma is one of the most aggressive solid tumors of the skin, caused by the malignant transformation of melanocytes. Melanoma is less common than non-melanoma skin cancers, but it is more severe because of its high metastasis rate and poor prognosis [109]. Approximately half of the patients with melanoma have BRAF mutations that result in constitutive ERK activation, thereby promoting the proliferation and migration of tumor cells [110]. Therefore, patients with BRAF mutations are regularly treated with a combination of BRAF and MEK (MAPK/ERK kinase) inhibitors. Because BRAF-mutated tumors also exhibit the overexpression of genes associated with immunosuppression, such as CTLA-4 or PD-L1, immune checkpoint inhibitors, anti-CTLA-4 and anti-PD-1 have also been approved for the treatment of melanoma [110]. However, novel approaches are needed to overcome the existing treatment resistance and prevent melanoma recurrence.

It has been found that the transformation of melanocytes to malignant melanoma cells is accompanied by changes in autophagy [111,112]. Malignant melanoma cells commonly display high levels of autophagy. A high autophagic index is associated with a poor response to treatment, poor survival, and aggressive tumor behavior [113]. In general, autophagy is considered to play a role in tumor suppression and promotion as cancer progresses. In the initial stages of tumorigenesis, autophagy acts as a tumor suppressor by reducing damaged cellular materials and limiting cell proliferation. In advanced stages, autophagy promotes the progression of established tumors by supplying nutrients that meet the metabolic demands of the cancer cells [114]. In melanoma, the overall role of autophagy is to help tumor cells survive in inappropriate microenvironment conditions [115,116]. Therefore, the inhibition of autophagy has become an interesting target to induce anti-tumor effects in advanced stages of melanoma [42]. Autophagy inhibition using chloroquine has been attempted in patients with malignant melanoma, but incomplete tumor eradication has been shown when this was implemented as a monotherapy [117]. As autophagy increases after the inhibition of oncoprotein signaling in several cancer types, the combined inhibition of autophagy and cancer type-specific signaling pathways has been conducted to elicit anti-tumor effects. BRAF inhibitors have been demonstrated to induce autophagy in melanomas bearing BRAF mutations, which provoke tumor proliferation and resistance to chemotherapy [118]. Thus, a combination of dabrafenib (BRAF inhibitor), trametinib (MEK inhibitor), and hydroxychloroquine (autophagy inhibitor) has been tested in patients with advanced melanoma bearing BRAF mutations [119].

## 8. Discussion

Autophagy is an intrinsic defense mechanism that maintains homeostasis in response to changes in the external environment. As the skin constitutes the outermost line of defense in contact with the external environment, the skin’s microenvironment is characterized by oxidative stress, invading pathogens, malnutrition, and so on. Therefore, autophagy is upregulated in most cell types located in the skin, including keratinocytes and immune cells. As keratinocytes are cells primarily exposed to external hazards that act as autophagy activators, autophagy is active in keratinocytes and participates in the differentiation and skin barrier function of keratinocytes. Immune cells that reside in the skin serve as an innate defense mechanism against invading pathogens, which activates and requires autophagy during antigen presentation and pathogen removal. Activation of autophagy in keratinocytes and skin immune cells is commonly mediated by core autophagy-related genes, including ATG5, ATG7, ULK1, and Beclin1, as we described in Section 3 and Section 4. In addition to the common autophagy components, each skin cell utilizes distinctive autophagy pathways (Figure 2). The skin cells require different kinds of selective autophagy: nucleophagy for keratinocytes, xenophagy for macrophages, and mitophagy for NK cells. Atg16L1 and TRIM5α mediate autophagic degradation of the Langerin-HIV-1 capsid complex in LCs. In macrophages and neutrophils, IL-1β secretion is mediated by autophagy. Collectively, skin cells not only share the core autophagy pathway, but also have a distinctive autophagy pathway depending on the cell type. As discussed in Section 5, abnormalities in the autophagy pathway are associated with the development of various skin diseases (Table 1). Decreased levels of autophagy-related proteins such as ATG4B and ATG5 and decreased autophagic degradation were reported in AA patient-derived HFs [69,70]. In contrast, an increase in autophagy-related protein levels was reported in PS and keloids [75,76,88,89,90]. These opposing results may arise because the studies measured the protein levels of Beclin-1 and LC3-II under steady-state conditions, not the autophagic flux. Since the increase in LC3-II levels in the steady state would result from the inhibition of autophagic degradation or the acceleration of autophagosome formation, the activity of autophagy should be measured by means of an autophagic flux assay [5]. In line with this, other studies have showed a decrease in autophagic degradation and autophagic flux or an increase in p62 protein levels in PS, AD, and keloids [30,77,78], suggesting that autophagy is inhibited in the disease models. The impaired autophagic degradation could be associated with lysosomal dysfunction, which has already been reported in Alzheimer’s disease, where autophagy acts as a main etiological mechanism. The downregulation of lysosomal enzymes—cathepsins—was observed in skin specimens from patients with AD or PS [77]. In addition, lysosomal acidification defects were observed in GAS-infected epithelial cells [102]. Thus, the reduction of autophagy activity or degradation in the skin cells may be a common pathogenesis of various skin diseases.

The involvement of autophagy in the inflammatory pathway has been reported in inflammatory skin diseases, such as AD and PS. IL-37 ameliorated allergic inflammation by stimulating the AMPK-mTOR-dependent autophagy pathway in a mouse model of AD [79]. The secretion of IL-1β and IL-36 was increased in keratinocytes derived from PS patients bearing AP1S3 mutations that inhibit autophagy [83,84]. In addition, human keloid fibroblasts exhibited reduced autophagic flux, resulting in elevated levels of the Notch1 protein, which is degraded by autophagy, and an increase in Notch-NLRP inflammasome formation [30]. These studies suggest that autophagy inducers may be promising therapeutics for skin diseases accompanied by inflammation.

Patients with patch-type AA show a good response to conventional immunosuppressants. In contrast, patients with alopecia totalis and alopecia universalis have chronic relapsing courses of disease and a poor response to treatment. It has been reported that local injection with platelet-rich plasma, stem cells, or their conditioned medium induces significant hair regrowth in patients with refractory AA by stimulating immunomodulatory effects and new anagen hair cycling. Stem cell therapy has a potent effect of inducing the differentiation of Treg cells and immune tolerance in many autoimmune diseases, including refractory AA. Treg cells inhibit the proinflammatory response of effector T cells by regulating the machinery of autophagy. As we described above, autophagy also affects the maintenance of active hair growth (anagen) during the hair cycle. Thus, the modulation of autophagy may lead to hair regrowth in patients with refractory AA by improving the HF immune privilege and active hair growth.

The modulation of autophagy in PS and AD may alleviate the expression of inflammatory cytokines and allergic inflammation. Both diseases have defects in skin barrier function, epidermal differentiation, and T-cell-mediated immune response, which is regulated by autophagy. Since Janus kinase (JAK) inhibitors and biologics were introduced to treat PS and AD, many patients have benefited from these treatments. Despite the great success of these drugs, the use of skin-barrier-strengthening moisturizers is still necessary. Moisturizers containing autophagy inducers that promote skin barrier function, epidermal differentiation, and anti-inflammation properties have been developed and can be useful as adjunctive treatments for patients with PS and AD.

Currently, there are only a few modalities used for keloid treatment in the clinical setting, such as intralesional triamcinolone injection, cryotherapy, and radiation therapy. The treatment is often unsatisfactory and accompanied by severe pain as a side effect. Thus, there is an unmet need for the development of new drugs based on the pathogenesis of keloids. Since autophagy also participates in wound healing by exerting anti-inflammatory and anti-infective activities in wounds and by facilitating the repair of damaged tissues, the modulation of autophagy may be beneficial in the treatment of keloids. Rapamycin, a well-known autophagy inducer, has been reported to have therapeutic effects on keloids, and other small molecules that enhance autophagy are currently under investigation.

## 9. Conclusions

Over the past two decades, there has been a substantial expansion in the knowledge regarding the molecular mechanisms of autophagy and its role in various diseases, including immune-related skin diseases. Since a basic knowledge of autophagy is a prerequisite to understanding advances in autophagy-related research fields, in this review, we have elucidated the roles of autophagy in various cell types in the skin and discussed its implications in immune-related skin diseases, such as inflammatory diseases, infectious diseases, and malignant melanoma. Autophagy, which is upregulated in the skin, has a beneficial effect on most skin cell types. Autophagy is essential for controlling the skin cell population, clearing invading pathogens, and stimulating anti-inflammatory activities. These processes require the core autophagy-related genes, such as ATG5 and Beclin-1, in most skin cells and unique autophagy subsets in specific skin cells, such as nucleophagy in keratinocytes and mitophagy in NK cells. Thus, autophagic impairment is thought to be critical in the pathogenesis of various immune-related skin diseases, including AA, PS, and melanoma. In other words, autophagy, like a guardian, supports skin cells in successfully performing their protective roles in maintaining skin homeostasis. Currently, autophagy inducers are being developed as treatments for various diseases, such as Alzheimer’s disease and cancer. However, there are some hurdles in the development of therapeutics because of drug delivery issues when targeting organs and side effects related to systemic treatment due to the involvement of autophagy in multiple signaling pathways. From this point of view, treatments for skin diseases have relatively fewer limitations. As the skin constitutes the outermost part of the body, topical drug application is available, avoiding side effects related to systemic drug administration. Therefore, autophagy modulation is a very attractive strategy for researchers looking for new potential treatments for skin diseases, although the given pathological conditions should be comprehensively considered. A combination of autophagy inducers with conventional therapies could represent a better strategy to minimize side effects and maximize therapeutic efficacy. Detailed insights into the function of autophagy in skin diseases may help to better understand its pathogenesis and lead to the development of more effective therapeutic approaches for these diseases.

## Figures and Tables

**Table 1:** Implication of autophagy in immune-related skin diseases.

Skin Diseases	Relevant Roles of Autophagy
Alopecia areata (AA)	Maintenance of the stage of growth (anagen) during the hair cycle and differentiation of hair follicle stem cells
Psoriasis (PS) and atopic dermatitis (AD)	Keratinocyte differentiation, skin barrier function, and inflammation prevention
Keloids	Notch1-NLRP3 inflammasome signaling activation and myofibroblast differentiation
Infectious disease	Pathogen elimination (anti-viral pathway) or viral replication and exit from the host (pro-viral pathway)
Melanoma	Tumor suppression by reducing harmful materials and limiting proliferation during initial stages and tumor promotion by supplying nutrients during advanced stages of tumorigenesis

## Data Availability

Not applicable.

## References

[1] Mizushima N., Komatsu M. (2011). Autophagy: Renovation of cells and tissues. Cell.

[2] Richmond J.M., Harris J.E. (2014). Immunology and skin in health and disease. Cold Spring Harb. Perspect. Med..

[3] Park H., Kang J.-H., Lee S. (2020). Autophagy in neurodegenerative diseases: A hunter for aggregates. Int. J. Mol. Sci..

[4] Scrivo A., Bourdenx M., Pampliega O., Cuervo A.M. (2018). Selective autophagy as a potential therapeutic target for neurodegenerative disorders. Lancet Neurol..

[5] Klionsky D.J., Abdalla F.C., Abeliovich H., Abraham R.T., Acevedo-Arozena A., Adeli K., Agholme L., Agnello M., Agostinis P., Aguirre-Ghiso J.A. (2012). Guidelines for the use and interpretation of assays for monitoring autophagy. Autophagy.

[6] Zhang X.-j., Chen S., Huang K.-x., Le W.-d. (2013). Why should autophagic flux be assessed?. Acta Pharmacol. Sin..

[7] Rogov V., Dötsch V., Johansen T., Kirkin V. (2014). Interactions between autophagy receptors and ubiquitin-like proteins form the molecular basis for selective autophagy. Mol. Cell.

[8] Di Rienzo M., Romagnoli A., Antonioli M., Piacentini M., Fimia G.M. (2020). TRIM proteins in autophagy: Selective sensors in cell damage and innate immune responses. Cell Death Differ..

[9] Menzies F.M., Fleming A., Caricasole A., Bento C.F., Andrews S.P., Ashkenazi A., Füllgrabe J., Jackson A., Sanchez M.J., Karabiyik C. (2017). Autophagy and neurodegeneration: Pathogenic mechanisms and therapeutic opportunities. Neuron.

[10] Dooley H.C., Razi M., Polson H.E., Girardin S.E., Wilson M.I., Tooze S.A. (2014). WIPI2 links LC3 conjugation with PI3P, autophagosome formation, and pathogen clearance by recruiting Atg12–5-16L1. Mol. Cell.

[11] Matoba K., Kotani T., Tsutsumi A., Tsuji T., Mori T., Noshiro D., Sugita Y., Nomura N., Iwata S., Ohsumi Y. (2020). Atg9 is a lipid scramblase that mediates autophagosomal membrane expansion. Nat. Struct. Mol. Biol..

[12] Itakura E., Kishi-Itakura C., Mizushima N. (2012). The hairpin-type tail-anchored SNARE syntaxin 17 targets to autophagosomes for fusion with endosomes/lysosomes. Cell.

[13] Chen Y., Yu L. (2017). Recent progress in autophagic lysosome reformation. Traffic.

[14] Ponpuak M., Mandell M.A., Kimura T., Chauhan S., Cleyrat C., Deretic V. (2015). Secretory autophagy. Curr. Opin. Cell Biol..

[15] Jiang S., Dupont N., Castillo E.F., Deretic V. (2013). Secretory versus degradative autophagy: Unconventional secretion of inflammatory mediators. J. Innate Immun..

[16] New J., Thomas S.M. (2019). Autophagy-dependent secretion: Mechanism, factors secreted, and disease implications. Autophagy.

[17] Jeong D., Qomaladewi N.P., Lee J., Park S.H., Cho J.Y. (2020). The role of autophagy in skin fibroblasts, keratinocytes, melanocytes, and epidermal stem cells. J. Investig. Dermatol..

[18] Akinduro O., Sully K., Patel A., Robinson D.J., Chikh A., McPhail G., Braun K.M., Philpott M.P., Harwood C.A., Byrne C. (2016). Constitutive autophagy and nucleophagy during epidermal differentiation. J. Investig. Dermatol..

[19] Monteleon C.L., Agnihotri T., Dahal A., Liu M., Rebecca V.W., Beatty G.L., Amaravadi R.K., Ridky T.W. (2018). Lysosomes support the degradation, signaling, and mitochondrial metabolism necessary for human epidermal differentiation. J. Investig. Dermatol..

[20] Belleudi F., Purpura V., Caputo S., Torrisi M.R. (2014). FGF7/KGF regulates autophagy in keratinocytes: A novel dual role in the induction of both assembly and turnover of autophagosomes. Autophagy.

[21] Xie X., Dai H., Zhuang B., Chai L., Xie Y., Li Y. (2016). Exogenous hydrogen sulfide promotes cell proliferation and differentiation by modulating autophagy in human keratinocytes. Biochem. Biophys. Res. Commun..

[22] Mijaljica D., Devenish R.J. (2013). Nucleophagy at a glance. J. Cell Sci..

[23] Song X., Narzt M.S., Nagelreiter I.M., Hohensinner P., Terlecki-Zaniewicz L., Tschachler E., Grillari J., Gruber F. (2017). Autophagy deficient keratinocytes display increased DNA damage, senescence and aberrant lipid composition after oxidative stress in vitro and in vivo. Redox Biol..

[24] Sukseree S., Rossiter H., Mildner M., Pammer J., Buchberger M., Gruber F., Watanapokasin R., Tschachler E., Eckhart L. (2013). Targeted deletion of Atg5 reveals differential roles of autophagy in keratin K5-expressing epithelia. Biochem. Biophys. Res. Commun..

[25] Yoshihara N., Ueno T., Takagi A., Trejo J.A.O., Haruna K., Suga Y., Komatsu M., Tanaka K., Ikeda S. (2015). The significant role of autophagy in the granular layer in normal skin differentiation and hair growth. Arch. Dermatol. Res..

[26] Noguchi S., Honda S., Saitoh T., Matsumura H., Nishimura E., Akira S., Shimizu S. (2019). Beclin 1 regulates recycling endosome and is required for skin development in mice. Commun. Biol..

[27] Qiang L., Yang S., Cui Y.-H., He Y.-Y. (2021). Keratinocyte autophagy enables the activation of keratinocytes and fibroblastsand facilitates wound healing. Autophagy.

[28] An Y., Liu W., Xue P., Ma Y., Zhang L., Zhu B., Qi M., Li L., Zhang Y., Wang Q. (2018). Autophagy promotes MSC-mediated vascularization in cutaneous wound healing via regulation of VEGF secretion. Cell Death Dis..

[29] Wu X., Fleming A., Ricketts T., Pavel M., Virgin H., Menzies F.M., Rubinsztein D.C. (2016). Autophagy regulates Notch degradation and modulates stem cell development and neurogenesis. Nat. Commun..

[30] Lee S., Kim S.K., Park H., Lee Y.J., Park S.H., Lee K.J., Lee D.G., Kang H., Kim J.E. (2020). Contribution of autophagy-Notch1-mediated NLRP3 inflammasome activation to chronic inflammation and fibrosis in keloid fibroblasts. Int. J. Mol. Sci..

[31] Doebel T., Voisin B., Nagao K. (2017). Langerhans cells–the macrophage in dendritic cell clothing. Trends Immunol..

[32] Nguyen A.V., Soulika A.M. (2019). The dynamics of the skin’s immune system. Int. J. Mol. Sci..

[33] Dang A.T., Teles R.M., Liu P.T., Choi A., Legaspi A., Sarno E.N., Ochoa M.T., Parvatiyar K., Cheng G., Gilliet M. (2019). Autophagy links antimicrobial activity with antigen presentation in Langerhans cells. JCI Insight.

[34] Ribeiro C., Sarrami-Forooshani R., Setiawan L.C., Zijlstra-Willems E.M., van Hamme J.L., Tigchelaar W., van der Wel N.N., Kootstra N.A., Gringhuis S.I., Geijtenbeek T.B. (2016). Receptor usage dictates HIV-1 restriction by human TRIM5α in dendritic cell subsets. Nature.

[35] Galle-Treger L., Hurrell B.P., Lewis G., Howard E., Jahani P.S., Banie H., Razani B., Soroosh P., Akbari O. (2020). Autophagy is critical for group 2 innate lymphoid cell metabolic homeostasis and effector function. J. Allergy Clin. Immunol..

[36] Sil P., Suwanpradid J., Muse G., Gruzdev A., Liu L., Corcoran D.L., Willson C.J., Janardhan K., Grimm S., Myers P. (2020). Noncanonical autophagy in dermal dendritic cells mediates immunosuppressive effects of UV exposure. J. Allergy Clin. Immunol..

[37] Huang J., Canadien V., Lam G.Y., Steinberg B.E., Dinauer M.C., Magalhaes M.A., Glogauer M., Grinstein S., Brumell J.H. (2009). Activation of antibacterial autophagy by NADPH oxidases. Proc. Natl. Acad. Sci. USA.

[38] Watson R.O., Manzanillo P.S., Cox J.S. (2012). Extracellular, M. tuberculosis DNA targets bacteria for autophagy by activating the host DNA-sensing pathway. Cell.

[39] Watson R.O., Bell S.L., MacDuff D.A., Kimmey J.M., Diner E.J., Olivas J., Vance R.E., Stallings C.L., Virgin H.W., Cox J.S. (2015). The cytosolic sensor cGAS detects Mycobacterium tuberculosis DNA to induce type I interferons and activate autophagy. Cell Host Microbe.

[40] Saitoh T., Fujita N., Jang M.H., Uematsu S., Yang B.-G., Satoh T., Omori H., Noda T., Yamamoto N., Komatsu M. (2008). Loss of the autophagy protein Atg16L1 enhances endotoxin-induced IL-1β production. Nature.

[41] Shi C.-S., Shenderov K., Huang N.-N., Kabat J., Abu-Asab M., Fitzgerald K.A., Sher A., Kehrl J.H. (2012). Activation of autophagy by inflammatory signals limits IL-1β production by targeting ubiquitinated inflammasomes for destruction. Nat. Immunol..

[42] Lazova R., Klump V., Pawelek J. (2010). Autophagy in cutaneous malignant melanoma. J. Cutan. Pathol..

[43] Jablonski N.G., Chaplin G. (2010). Human skin pigmentation as an adaptation to UV radiation. Proc. Natl. Acad. Sci. USA.

[44] Liu Y., Hong L., Kempf V.R., Wakamatsu K., Ito S., Simon J.D. (2004). Ion-exchange and adsorption of Fe (III) by Sepia melanin. Pigment Cell Res..

[45] Ushio H., Ueno T., Kojima Y., Komatsu M., Tanaka S., Yamamoto A., Ichimura Y., Ezaki J., Nishida K., Komazawa-Sakon S. (2011). Crucial role for autophagy in degranulation of mast cells. J. Allergy Clin. Immunol..

[46] Wang B., Li X., Li M., Geng Y., Wang N., Jin Y., Zhang W., Xu K., Wang J., Tao L. (2022). Dopamine D3 receptor signaling alleviates mouse rheumatoid arthritis by promoting Toll-like receptor 4 degradation in mast cells. Cell Death Dis..

[47] Sanjuan M.A., Dillon C.P., Tait S.W., Moshiach S., Dorsey F., Connell S., Komatsu M., Tanaka K., Cleveland J.L., Withoff S. (2007). Toll-like receptor signalling in macrophages links the autophagy pathway to phagocytosis. Nature.

[48] Bhattacharya A., Wei Q., Shin J.N., Fattah E.A., Bonilla D.L., Xiang Q., Eissa N.T. (2015). Autophagy is required for neutrophil-mediated inflammation. Cell Rep..

[49] Iula L., Keitelman I.A., Sabbione F., Fuentes F., Guzman M., Galletti J.G., Gerber P.P., Ostrowski M., Geffner J.R., Jancic C.C. (2018). Autophagy mediates interleukin-1β secretion in human neutrophils. Front. Immunol..

[50] Yost C.C., Cody M.J., Harris E.S., Thornton N.L., McInturff A.M., Martinez M.L., Chandler N.B., Rodesch C.K., Albertine K.H., Petti C.A. (2009). Impaired neutrophil extracellular trap (NET) formation: A novel innate immune deficiency of human neonates. Blood J. Am. Soc. Hematol..

[51] Arai Y., Nishinaka Y., Arai T., Morita M., Mizugishi K., Adachi S., Takaori-Kondo A., Watanabe T., Yamashita K. (2014). Uric acid induces NADPH oxidase-independent neutrophil extracellular trap formation. Biochem. Biophys. Res. Commun..

[52] Sharma A., Simonson T.J., Jondle C.N., Mishra B.B., Sharma J. (2017). Mincle-mediated neutrophil extracellular trap formation by regulation of autophagy. J. Infect. Dis..

[53] Germic N., Stojkov D., Oberson K., Yousefi S., Simon H.U. (2017). Neither eosinophils nor neutrophils require ATG 5-dependent autophagy for extracellular DNA trap formation. Immunology.

[54] Wang S., Xia P., Huang G., Zhu P., Liu J., Ye B., Du Y., Fan Z. (2016). FoxO1-mediated autophagy is required for NK cell development and innate immunity. Nat. Commun..

[55] O’Sullivan T.E., Geary C.D., Weizman O.-E., Geiger T.L., Rapp M., Dorn G.W., Overholtzer M., Sun J.C. (2016). Atg5 is essential for the development and survival of innate lymphocytes. Cell Rep..

[56] Lei V., Petty A.J., Atwater A.R., Wolfe S.A., MacLeod A.S. (2020). Skin Viral Infections: Host Antiviral Innate Immunity and Viral Immune Evasion. Front. Immunol..

[57] Baginska J., Viry E., Berchem G., Poli A., Noman M.Z., van Moer K., Medves S., Zimmer J., Oudin A., Niclou S.P. (2013). Granzyme B degradation by autophagy decreases tumor cell susceptibility to natural killer-mediated lysis under hypoxia. Proc. Natl. Acad. Sci. USA.

[58] Mgrditchian T., Arakelian T., Paggetti J., Noman M.Z., Viry E., Moussay E., Van Moer K., Kreis S., Guerin C., Buart S. (2017). Targeting autophagy inhibits melanoma growth by enhancing NK cells infiltration in a CCL5-dependent manner. Proc. Natl. Acad. Sci. USA.

[59] Clark R.A. (2010). Skin-resident T cells: The ups and downs of on site immunity. J. Investig. Dermatol..

[60] Le Texier L., Lineburg K.E., Cao B., McDonald-Hyman C., Leveque-El Mouttie L., Nicholls J., Melino M., Nalkurthi B.C., Alexander K.A., Teal B. (2016). Autophagy-dependent regulatory T cells are critical for the control of graft-versus-host disease. JCI Insight.

[61] Alissafi T., Banos A., Boon L., Sparwasser T., Ghigo A., Wing K., Vassilopoulos D., Boumpas D., Chavakis T., Cadwell K. (2017). Tregs restrain dendritic cell autophagy to ameliorate autoimmunity. J. Clin. Investig..

[62] Wang X., Hu C., Wu X., Wang S., Zhang A., Chen W., Shen Y., Tan R., Wu X., Sun Y. (2016). Roseotoxin B improves allergic contact dermatitis through a unique anti-inflammatory mechanism involving excessive activation of autophagy in activated T lymphocytes. J. Investig. Dermatol..

[63] Suchonwanit P., Kositkuljorn C., Pomsoong C. (2021). Alopecia Areata: An Autoimmune Disease of Multiple Players. Immuno Targets Ther..

[64] Stenn K.S., Paus R. (2001). Controls of hair follicle cycling. Physiol. Rev..

[65] Subramanya R.D., Coda A.B., Sinha A.A. (2010). Transcriptional profiling in alopecia areata defines immune and cell cycle control related genes within disease-specific signatures. Genomics.

[66] Parodi C., Hardman J.A., Allavena G., Marotta R., Catelani T., Bertolini M., Paus R., Grimaldi B. (2018). Autophagy is essential for maintaining the growth of a human (mini-)organ: Evidence from scalp hair follicle organ culture. PLoS Biol..

[67] Cai B., Zheng Y., Yan J., Wang J., Liu X., Yin G. (2019). BMP2-mediated PTEN enhancement promotes differentiation of hair follicle stem cells by inducing autophagy. Exp. Cell Res..

[68] Betz R.C., Petukhova L., Ripke S., Huang H., Menelaou A., Redler S., Becker T., Heilmann S., Yamany T., Duvic M. (2015). Genome-wide meta-analysis in alopecia areata resolves HLA associations and reveals two new susceptibility loci. Nat. Commun..

[69] Petukhova L., Patel A.V., Rigo R.K., Bian L., Verbitsky M., Sanna-Cherchi S., Erjavec S.O., Abdelaziz A.R., Cerise J.E., Jabbari A. (2020). Integrative analysis of rare copy number variants and gene expression data in alopecia areata implicates an aetiological role for autophagy. Exp. Dermatol..

[70] Hardman J.A., Nicu C., Tai C., Harries M., Mironov A., Purba T.S., Paus R. (2020). Does dysfunctional autophagy contribute to immune privilege collapse and alopecia areata pathogenesis?. J. Dermatol. Sci..

[71] Mosca M., Hong J., Hadeler E., Hakimi M., Liao W., Bhutani T. (2021). The Role of IL-17 Cytokines in Psoriasis. Immuno Targets Ther..

[72] Bovenschen H.J., van de Kerkhof P.C., van Erp P.E., Woestenenk R., Joosten I., Koenen H.J. (2011). Foxp3+ regulatory T cells of psoriasis patients easily differentiate into IL-17A-producing cells and are found in lesional skin. J. Investig. Dermatol..

[73] Brunner P.M., Guttman-Yassky E., Leung D.Y. (2017). The immunology of atopic dermatitis and its reversibility with broad-spectrum and targeted therapies. J. Allergy Clin. Immunol..

[74] Peters N., Peters A.T. (2019). Atopic dermatitis. Allergy Asthma Proc..

[75] Amer A.S., Samaka R.M., Moftah N.H. (2021). Beclin1 in psoriasis: An immunohistochemical study. Clin. Exp. Dermatol..

[76] Karabowicz P., Wronski A., Ostrowska H., Waeg G., Zarkovic N., Skrzydlewska E. (2020). Reduced Proteasome Activity and Enhanced Autophagy in Blood Cells of Psoriatic Patients. Int. J. Mol. Sci..

[77] Klapan K., Frangez Z., Markov N., Yousefi S., Simon D., Simon H.U. (2021). Evidence for Lysosomal Dysfunction within the Epidermis in Psoriasis and Atopic Dermatitis. J. Investig. Dermatol..

[78] Hailfinger S., Schulze-Osthoff K. (2021). Impaired Autophagy in Psoriasis and Atopic Dermatitis: A New Therapeutic Target?. J. Investig. Dermatol..

[79] Hou T., Sun X., Zhu J., Hon K.L., Jiang P., Chu I.M., Tsang M.S., Lam C.W., Zeng H., Wong C.K. (2020). IL-37 Ameliorating Allergic Inflammation in Atopic Dermatitis Through Regulating Microbiota and AMPK-mTOR Signaling Pathway-Modulated Autophagy Mechanism. Front. Immunol..

[80] Nakahira K., Haspel J.A., Rathinam V.A., Lee S.J., Dolinay T., Lam H.C., Englert J.A., Rabinovitch M., Cernadas M., Kim H.P. (2011). Autophagy proteins regulate innate immune responses by inhibiting the release of mitochondrial DNA mediated by the NALP3 inflammasome. Nat. Immunol..

[81] Harris J., Hartman M., Roche C., Zeng S.G., O’Shea A., Sharp F.A., Lambe E.M., Creagh E.M., Golenbock D.T., Tschopp J. (2011). Autophagy controls IL-1beta secretion by targeting pro-IL-1beta for degradation. J. Biol. Chem..

[82] Kwon S.H., Lim C.J., Jung J., Kim H.J., Park K., Shin J.W., Huh C.H., Park K.C., Na J.I. (2019). The effect of autophagy-enhancing peptide in moisturizer on atopic dermatitis: A randomized controlled trial. J. Dermatol. Treat..

[83] Setta-Kaffetzi N., Simpson M.A., Navarini A.A., Patel V.M., Lu H.-C., Allen M.H., Duckworth M., Bachelez H., Burden A.D., Choon S.-E. (2014). AP1S3 mutations are associated with pustular psoriasis and impaired Toll-like receptor 3 trafficking. Am. J. Hum. Genet..

[84] Mahil S.K., Twelves S., Farkas K., Setta-Kaffetzi N., Burden A.D., Gach J.E., Irvine A.D., Képíró L., Mockenhaupt M., Oon H.H. (2016). AP1S3 mutations cause skin autoinflammation by disrupting keratinocyte autophagy and up-regulating IL-36 production. J. Investig. Dermatol..

[85] Douroudis K., Kingo K., Traks T., Reimann E., Raud K., Ratsep R., Mossner R., Silm H., Vasar E., Koks S. (2012). Polymorphisms in the ATG16L1 gene are associated with psoriasis vulgaris. Acta Derm. Venereol..

[86] Betarbet U., Blalock T.W. (2020). Keloids: A Review of Etiology, Prevention, and Treatment. J. Clin. Aesthetic Dermatol..

[87] Nakashima M., Chung S., Takahashi A., Kamatani N., Kawaguchi T., Tsunoda T., Hosono N., Kubo M., Nakamura Y., Zembutsu H. (2010). A genome-wide association study identifies four susceptibility loci for keloid in the Japanese population. Nat. Genet..

[88] Jeon Y.R., Roh H., Jung J.H., Ahn H.M., Lee J.H., Yun C.O., Lee W.J. (2019). Antifibrotic Effects of High-Mobility Group Box 1 Protein Inhibitor (Glycyrrhizin) on Keloid Fibroblasts and Keloid Spheroids through Reduction of Autophagy and Induction of Apoptosis. Int. J. Mol. Sci..

[89] Okuno R., Ito Y., Eid N., Otsuki Y., Kondo Y., Ueda K. (2018). Upregulation of autophagy and glycolysis markers in keloid hypoxic-zone fibroblasts: Morphological characteristics and implications. Histol. Histopathol..

[90] Wang Q., Wang P., Qin Z., Yang X., Pan B., Nie F., Bi H. (2021). Altered glucose metabolism and cell function in keloid fibroblasts under hypoxia. Redox Biol..

[91] Ong C.T., Khoo Y.T., Mukhopadhyay A., Do D.V., Lim I.J., Aalami O., Phan T.T. (2007). mTOR as a potential therapeutic target for treatment of keloids and excessive scars. Exp. Dermatol..

[92] Coates M., Blanchard S., MacLeod A.S. (2018). Innate antimicrobial immunity in the skin: A protective barrier against bacteria, viruses, and fungi. PLoS Pathog..

[93] Shoji-Kawata S., Levine B. (2009). Autophagy, antiviral immunity, and viral countermeasures. Biochim. Biophys. Acta.

[94] Tam J.M., Mansour M.K., Acharya M., Sokolovska A., Timmons A.K., Lacy-Hulbert A., Vyas J.M. (2016). The Role of Autophagy-Related Proteins in Candida albicans Infections. Pathogens.

[95] Duarte L.F., Reyes A., Farias M.A., Riedel C.A., Bueno S.M., Kalergis A.M., Gonzalez P.A. (2021). Crosstalk between Epithelial Cells, Neurons and Immune Mediators in HSV-1 Skin Infection. Front. Immunol..

[96] O’Connell D., Liang C. (2016). Autophagy interaction with herpes simplex virus type-1 infection. Autophagy.

[97] Kennedy P.G.E., Mogensen T.H., Cohrs R.J. (2021). Recent Issues in Varicella-Zoster Virus Latency. Viruses.

[98] Clebak K.T., Malone M.A. (2018). Skin infections. Prim. Care Clin. Off. Pract..

[99] Hansen N.S., Leth S., Nielsen L.T. (2020). Toksisk shock-syndrom. Ugeskr. Laeger.

[100] Nakagawa I., Amano A., Mizushima N., Yamamoto A., Yamaguchi H., Kamimoto T., Nara A., Funao J., Na-kata M., Tsuda K. (2004). Autophagy defends cells against invading group A Streptococcus. Science.

[101] Lin C.Y., Nozawa T., Minowa-Nozawa A., Toh H., Hikichi M., Iibushi J., Nakagawa I. (2020). Autophagy Receptor Tollip Facilitates Bacterial Autophagy by Recruiting Galectin-7 in Response to Group A Streptococcus Infection. Front. Cell. Infect. Microbiol..

[102] Lu S.L., Kuo C.F., Chen H.W., Yang Y.S., Liu C.C., Anderson R., Wu J.J., Lin Y.S. (2015). Insufficient Acidification of Autophagosomes Facilitates Group A Streptococcus Survival and Growth in Endothelial Cells. mBio.

[103] Barnett T.C., Liebl D., Seymour L.M., Gillen C.M., Lim J.Y., Larock C.N., Davies M.R., Schulz B.L., Nizet V., Teasdale R.D. (2013). The globally disseminated M1T1 clone of group A Streptococcus evades autophagy for intracellular replication. Cell Host Microbe.

[104] Surewaard B.G., Deniset J.F., Zemp F.J., Amrein M., Otto M., Conly J., Omri A., Yates R.M., Kubes P. (2016). Identification and treatment of the *Staphylococcus aureus* reservoir in vivo. J. Exp. Med..

[105] Wang M., Fan Z., Han H. (2021). Autophagy in *Staphylococcus aureus* Infection. Front. Cell. Infect. Microbiol..

[106] Franco-Paredes C., Marcos L.A., Henao-Martínez A.F., Rodríguez-Morales A.J., Villamil-Gómez W.E., Gotuzzo E., Bonifaz A. (2018). Cutaneous mycobacterial infections. Clin. Microbiol. Rev..

[107] Griffith D.E., Aksamit T., Brown-Elliott B.A., Catanzaro A., Daley C., Gordin F., Holland S.M., Horsburgh R., Huitt G., Iademarco M.F. (2007). An official ATS/IDSA statement: Diagnosis, treatment, and prevention of nontuberculous mycobacterial diseases. Am. J. Respir. Crit. Care Med..

[108] Silwal P., Kim I.S., Jo E.K. (2021). Autophagy and Host Defense in Nontuberculous Mycobacterial Infection. Front. Immunol..

[109] DiGiacinto D., Bagley J., Goldsbury A.M. (2016). The Value of Sonography in the Assessment of Skin Cancers and Their Metastases. J. Diagn. Med. Sonogr..

[110] Jung T., Haist M., Kuske M., Grabbe S., Bros M. (2021). Immunomodulatory Properties of BRAF and MEK Inhibitors Used for Melanoma Therapy-Paradoxical ERK Activation and Beyond. Int. J. Mol. Sci..

[111] Levine B., Kroemer G. (2008). Autophagy in the pathogenesis of disease. Cell.

[112] Rosenfeldt M.T., Ryan K.M. (2011). The multiple roles of autophagy in cancer. Carcinogenesis.

[113] Ma X.H., Piao S., Wang D., McAfee Q.W., Nathanson K.L., Lum J.J., Li L.Z., Amaravadi R.K. (2011). Measurements of tumor cell autophagy predict invasiveness, resistance to chemotherapy, and survival in melanoma. Clin. Cancer Res..

[114] Yun C.W., Lee S.H. (2018). The Roles of Autophagy in Cancer. Int. J. Mol. Sci..

[115] Rahmati M., Ebrahim S., Hashemi S., Motamedi M., Moosavi M.A. (2020). New insights on the role of autophagy in the pathogenesis and treatment of melanoma. Mol. Biol. Rep..

[116] Di Leo L., Bodemeyer V., De Zio D. (2020). The complex role of autophagy in melanoma evolution: New perspectives from mouse models. Front. Oncol..

[117] Foth M., McMahon M. (2021). Autophagy Inhibition in BRAF-Driven Cancers. Cancers.

[118] Li S., Song Y., Quach C., Guo H., Jang G.B., Maazi H., Zhao S., Sands N.A., Liu Q., In G.K. (2019). Transcriptional regulation of autophagy-lysosomal function in BRAF-driven melanoma progression and chemoresistance. Nat. Commun..

[119] Mehnert J.M., Mitchell T.C., Huang A.C., Aleman T.S., Kim B.J., Schuchter L.M., Linette G.P., Karakousis G.C., Mitnick S., Giles L. (2022). BAMM (BRAF Autophagy and MEK Inhibition in Melanoma): A Phase I/II Trial of Dabrafenib, Trametinib, and Hydroxychloroquine in Advanced BRAFV600-mutant Melanoma. Clin. Cancer Res..

